# Immunomodulatory treatment for lymphocytic myocarditis—a systematic review and meta-analysis

**DOI:** 10.1007/s10741-018-9709-9

**Published:** 2018-06-04

**Authors:** Max-Paul Winter, Patrick Sulzgruber, Lorenz Koller, Philipp Bartko, Georg Goliasch, Alexander Niessner

**Affiliations:** Department of Internal Medicine II, Medical University of Vienna, Waehringer Guertel 18-20, 1090 Vienna, Austria

**Keywords:** Myocarditis, Inflammatory cardiomyopathy, Immunosuppression, Immunomodulation

## Abstract

Deleterious inflammatory responses are seen to be the trigger of heart failure in myocarditis and therapies directed towards immunomodulation have been assumed to be beneficial. The objective of the present review was to systematically assess the effect of immunomodulation in lymphocytic myocarditis. Studies were included if diagnosis of lymphocytic myocarditis was based on EMB as well as on the exclusion of other etiologies of heart failure and if the patients had at least moderately decreased left ventricular ejection fraction (< 45%). All immunomodulatory treatments at any dose that target the cause of myocarditis leading to cardiomyopathy were included. Retrieval of PUBMED, SCOPUS, Cochrane Central Register of Controlled Trials, and LILACs from January 1950 to January 2016 revealed 444 abstracts of which nine studies with a total of 612 patients were included. As primary effectivity endpoint, a change in left ventricular ejection was chosen. No benefits of corticosteroids or intravenous immunoglobulin alone were reported. Immunoadsorption and subsequent IVIG substitution was associated with a greater improvement in left ventricular ejection fraction (LVEF) in one study. Single studies found a beneficial effect of interferon and statins on LVEF. We performed a meta-analysis for the combination of corticosteroids with immunosuppressants and found a non-significant increase of LVEF of + 13.06% favoring combined treatment (95%CI 1.71 to + 27.84%, *p =* 0.08). The current evidence does not support the routine use of immunosuppression in traditional lymphocytic myocarditis. Nevertheless, in histologically proven virus-negative myocarditis of high-risk patients, combined immunosuppression might be beneficial. Future research should focus on translation of these effects to clinical outcome.

## Introduction

Myocarditis is defined as an inflammatory disease of the myocardium, histopathologically depicted by infiltration of mononuclear cells to the heart muscle with the presence of myocellular necrosis [1, 2]. Clinical presentation is heterogeneous, ranging from subclinical disease with asymptomatic ECG changes to sudden cardiac death and acute deteriorating heart failure. The exclusion of other causes for heart failure and combination of symptoms, laboratory testing, ECG findings, and cardiac imaging leads to the diagnosis of myocarditis [3, 4]. Endomyocardial biopsy (EMB) has shown that viral infections are the most important causes of myocarditis with up to 38% of the samples being positive for viral genomes [5–7]. Beside the infectious agent per se, the maladaptive immune-mediated responses against these agents are seen to be causative for the myocardial cell dysfunction and compromised contractility [3, 5]. Outcome in myocarditis largely depends the development of malignant arrhythmias [8, 9], acute heart failure [8], as well as the development of chronic active myocarditis and dilative cardiomyopathy (DCM) [10]. Determinants of progression from myocarditis to DCM remain unknown, but predominantly occur in patients with failure of viral clearance leading to persistent inflammation or those that develop pathogenic cardiac autoantibodies directed against myocardial epitopes [11, 12]. To date, treatment of myocarditis largely focuses on supportive care to prevent heart failure or concomitant adverse events; however, no approved curative therapy is available. As deleterious inflammatory responses to viral infections provoke myocardial dysfunction in myocarditis, therapies directed towards immunomodulation have been assumed to be beneficial. The scope of this review is these curative therapies with immunomodulatory effect in lymphocytic myocarditis. Due to the high incidence of spontaneous improvement in left ventricular function and the responsiveness to conventional heart failure treatment, only randomized trials with a properly defined control arm that truly evaluates efficiency of the treatment were analyzed [3]. Owing to the rare incidence of myocarditis with severely impaired left ventricular function, this systematic review will assess studies assessing the objective surrogate endpoint of echocardiographic measurement of left ventricular function.

## Methods

The reported search strategy, study selection, data extraction, and analysis were performed according to the PRISMA guidelines for systematic reviews and meta-analysis [13].

### Search strategy

Two authors (M.W. and P.S.) systematically searched PUBMED, SCOPUS, Cochrane Central Register of Controlled Trials, and LILACs for eligible trials from January 1950 to January 2016. To prevent potential publication bias, trial registries (www.who.int/trialsearch/Default.aspx, the WHO International Clinical Trials Registry Platform, and www.clinicaltrials.gov) were screened for ongoing and completed trials. The search strategy was based on the combination of disease, therapy, and study design using “AND” and “OR.”

### Definitions and interventions

Studies were only eligible if participants were diagnosed with traditional myocarditis. Diagnosis of myocarditis was based on EMB and the exclusion of other etiologies of heart failure [8]. Patients in the included trials had at least moderately decreased left ventricular ejection fraction (LVEF) (< 45%). Studies including patients with different etiologies of heart failure were included only if there was a separate analysis of the results for patients with myocarditis. Studies investigating etiologies of the disease other than traditional lymphocytic myocarditis (specific pathogens such as *Borrelia burgdorferi* and *Trypanosoma cruzi*, cardiac sarcoidosis) were excluded [14–16].

Due to the multifactorial etiology of peripartum cardiomyopathy (angiogenic imbalance [17], altered prolactin processing [18], inflammatory cytokines [19], and myocarditis [20]) and difficulties to treat as per protocol given by the pregnancy, patients with peripartum cardiomyopathy were not included into the clinical trials and could not be included in this analysis.

All immunomodulatory treatments at any dose that target the cause of myocarditis leading to cardiomyopathy were included.

### Study selection and data extraction

All published studies investigating curative immunomodulatory treatment to prevent the development of cardiomyopathy as a sequel of myocarditis were identified. M.W. and P.S. screened titles and/or abstracts for inclusion and in a second step, all potentially suitable manuscripts were reviewed for final eligibility. Duplicates were identified using the reference management software EndNote X6 (Thomson Reuters, NY, USA) and excluded. Additionally, the reference lists of the included articles and reviews were examined for further relevant publications. Only randomized and controlled trials were included. Full texts of all includable trials were obtained and two investigators (M.W. and A.N.) independently assessed study eligibility and extracted the data. The following details were recorded for each study: the first author, study design, patient characteristics, inclusion criteria, interventions, outcome measurements, and funding.

### Outcomes and measurements

The primary efficacy outcome was LVEF measured by echocardiography or scintigraphy. Secondary efficacy endpoints were (i) New York Heart Association (NYHA) functional classification and (ii) viral clearance and resolution of the inflammatory infiltrate in the myocardium.

### Study quality assessment

Quality of included studies was assessed according to the Cochrane Handbook for Systematic Reviews of Interventions 5.1.0 [21]. Two investigators (M.W. and A.N.) evaluated methodological quality of the studies independently. Studies with “inadequate” methodology were excluded. Furthermore, blinding of those providing and receiving the intervention (double blinding), description of losses to follow-up, and the use of intention-to-treat analysis were documented. Disagreements were resolved by consensus.

### Data analysis

All results are summarized as mean difference for continuous variables or risk ratio for dichotomous variables and 95% confidence interval (CI). Due to the heterogeneity of the different treatments of myocarditis, an overall meta-analysis was not feasible. Meta-analysis was performed for therapies with ≥ 3 studies assessing the effect on the primary surrogate endpoint LVEF. A test for heterogeneity was used to decide whether a fixed effects model or a random effects model is adequate. Before entering into the meta-analysis, extracted data is transformed from standard error of the mean (SEM) or CI to standard deviation (SD). The potential for publication bias was assessed using a funnel plot. Statistical analysis was performed using the program Review Manager 5.0 (RevMan, Copenhagen: The Nordic Cochrane Centre, The Cochrane Collaboration, 2008).

## Results

### Study selection and characteristics

**Fig. 1:**
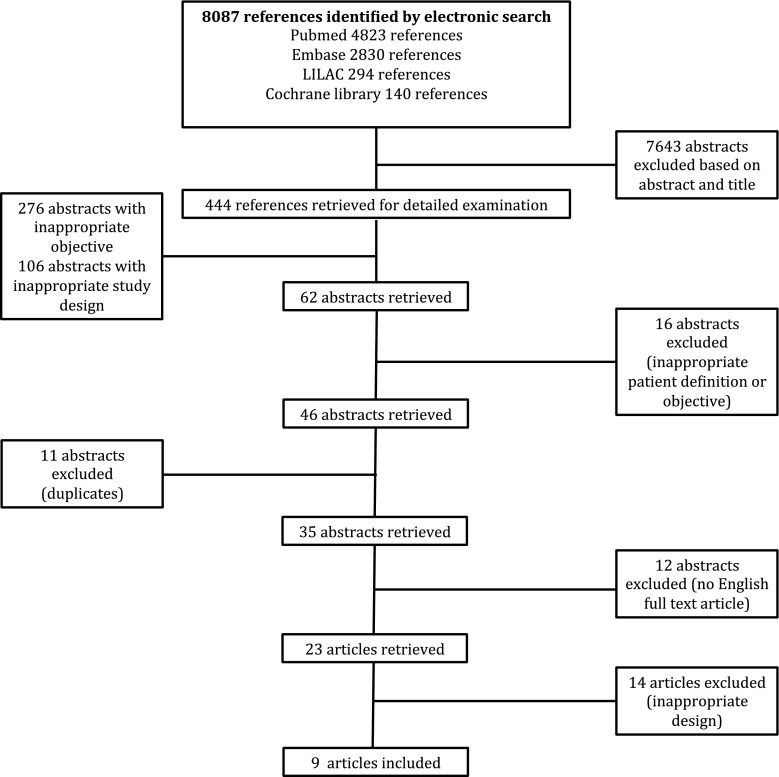
The flow diagram of study selection

The database search on PUBMED, EMBASE, Cochrane Central Register of Controlled Trials, and LILAC retrieved 444 abstracts. Three hundred eighty-two abstracts were excluded because of an inappropriate objective or study design. From the remaining abstracts, 11 duplicates were excluded, 16 abstracts were excluded because of inappropriate patient or objective definition, and 12 abstracts were not available in English and were excluded. Overall, 23 full-text articles were retrieved. Fourteen articles were excluded because of inappropriate study design or inadequate definition of myocarditis. Inter-readers agreement was high (kappa coefficient 0.99). Nine controlled trials randomized 612 patients to immunomodulatory therapy versus standard therapy ± placebo (Fig. 1). The main characteristics of the included studies are listed in Table 1.

**Table 1:** Characteristics of the included studies

Study ID	Design	Intervention	Placebo	No. of patients	Age	Disease	Duration of symptom	NYHA baseline	Spec. inclusion criteria	Exclusion	BX	LV baseline
Parillo 1989 [22]	Randomized, controlled	Prednisone	No	102	43 (23–67)	DCM	< 2 years, mean 8 m	NA	None	CAD, HTN, valvular CM, congenital CM	Yes	< 35%
Latham 1989 [23]	Randomized, controlled	Prednisone	No	52	41 ± 12 mean ± SD	DCM	< 2 years, mean: 1.6–1.8 m	II–IV	None	CAD, HTN, DMII, secondary CM	Yes	< 40%
Mason 1995 [24]	Randomized, controlled, open-label multicenter	Azathioprine or cyclosporine + prednisone	No	111	Int: 43 ± 14 Cont: 41 ± 13 mean ± SD	MYO	< 2 years; 43–51% < 1 m	I–IV	Myocardial inflammation	CAD	Yes	< 45%
Miric 1996 [25]	Randomized, controlled, open label	Interferon-α or thymomodulin	No	38	10–54 range	DCM or MYO	NA	II–IV	Myocardial inflammation	CAD, valvular CM, HTN, post-partum CM, congenital CM, giant-cell MYO	Yes	< 45
Wojnicz 2001 [26]	Randomized, controlled, open-label	Azathioprine and prednisone	Yes	84	Int: 41 (16–61) Cont: 39 (29–60) Mean (95% CI)	DCM	> 6 m	II–IV	Increased HLA expression	CAD, HTN, valvular CM/other CM	Yes	< 40%
Staudt 2001 [27]	Randomized, controlled	Immunoadsorption + IgG substitution	No	25	Int: 50 ± 3 Cont: 50 ± 3 mean ± SEM	DCM	< 6 m	III–IV	Myocardial inflammation		Yes	< 30%
Wojnicz 2006 [28]	Randomized, controlled, open label, 2 center study	Atorvastatin	No	74	39 ± 12 mean ± SD	DCM	> 6 m	II–III	HLA upregulation	CAD	Yes	< 40%
Frustaci 2009 [29]	Randomized, controlled, double blinded, multicenter	Azathioprine and prednisone	Yes	85	Int: 44.2 ± 15.8 Cont: 41.1 ± 15.1 mean ± SD	DCM or MYO	> 6 m	II–IV	Myocardial inflammation	Less than 6 months onset of heart failure; CAD, sec. CMP, endocrine disease, significant CNI drug or alcohol abuse]; (iii) therapy with steroids within 6 months before the enrolment	Yes	< 45%
Kishimoto 2014 [30]	Randomized, controlled, multicenter	IVIG	No	41	19–76 range	DCM or MYO	> 6 m	III–IV	None	DM II, thyroid disease, renal disease, uncontrolled hypertension valvular heart disease, CAD	Yes (50%)	< 40%

*Int* intervention group, *Cont* control group, *NA* not available, *CM* cardiomyopathy, *DCM* dilated cardiomyopathy, *NICM* non-ischemic cardiomyopathy, *CAD* coronary artery disease, *MI* myocardial infarction, *UA* unstable angina, *ACS* acute coronary syndrome, *MYO* myocarditis, *DM* diabetes mellitus, *HTN* hypertension, *m* months

Endomyocardial biopsy at baseline was performed in all studies as demanded per protocol. With the exception of one study that did not report the duration of symptoms (or duration of heart failure treatment), the duration at baseline was at least 3 months in all studies and was limited to 6 months to 2 years in seven studies.

Studies assessed immunomodulatory treatment with corticosteroids only (*n* = 2) [22, 23], a combination of immunosuppressants and corticosteroids (*n* = 3) [24, 26, 29], intravenous immunoglobulin (IVIG) only (*n* = 1) [30], a combination of immunoadsorption and IVIG (*n* = 1) [27], statin (*n* = 1) [28], and anti-viral treatment with interferon or the interferon-inducing agent thymomodulin (*n* = 1) [25]. Two studies applied a placebo treatment in the control group (Fig. 3).

### Prednisone and immunosuppressive drugs

Prednisone has been tested as monotherapy or in combination with other immunosuppressants. Regarding monotherapy, two studies used prednisone over 3 months. Neither study found any significant differences of LVEF, NYHA functional class, inflammation of the myocardium, nor number of clinical events over a follow-up period of 15 to 24 months [22, 23]. Parillo et al. reported a discrete improvement of the LVEF after 3 months in the prednisone group (*n* = 49) from 17.9 ± 1.0 to 22.2 ± 1.0 versus 17.1 ± 1.1 to 19.3 ± 1.4% in the control group (*n* = 52), but this difference failed to reach statistical significance [22].

Mason et al., Wojnicz et al., and Frustaci et al. investigated the effect of a combined treatment with an immunosuppressant (cyclosporine or azathioprine) and prednisone for 3 to 6 months [24, 26, 29]. Wojnicz et al. and Frustaci et al. found a significantly higher LVEF in the treatment group within the observational periods. Wojnicz et al. reported in the immunosuppression group (*n* = 41) an increase of LVEF from 23.8 ± 8.6 to 35.9 ± 10.0% as compared to the control group (*n* = 43) from 24.9 ± 7.3 to 27.2 ± 10.1% (*p* < 0.001). In line with this observation, Frustaci et al. found in the immunosuppression group (*n* = 43) an increase of LVEF from 26.5 ± 6.7 to 45.6 ± 9.6% as compared to a change from 27.7 ± 5.6 to 21.3 ± 5.3% (*p* < 0.001) in the control group (*n* = 42).

**Fig. 2:**
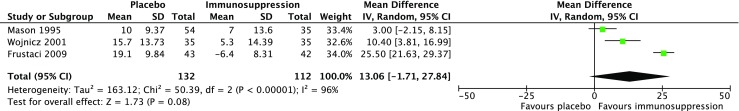
Forrest plot and meta-analysis for the effect of combined immunosuppression on ventricular ejection fraction. Results from the latest follow-up time point were used. Raw data were transformed to mean ± SD and the mean difference (95% CI) of left ventricular ejection fraction (%) between treatment groups was calculated with the Review Manager

**Fig. 3:**
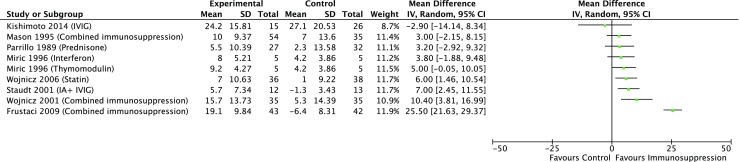
Forrest plot for the effect of all published immunomodulatory treatments on ventricular ejection fraction. Results from the latest follow-up time point were used. Raw data were transformed to mean ± SD and the mean difference (95% CI) of left ventricular ejection fraction (%) between treatment groups was calculated with the Review Manager. IA, immunoadsorption; IVIG, immunoglobulins

This improvement was accompanied by an improvement of symptoms as depicted by a significant decrease in NYHA functional class. In contrast to these data, Mason et al. found no significant differences in changes of the LVEF between treatment and control groups at 28 and 52 weeks [24]. The pooled difference of the increase of LVEF between the combined immunosuppression and control groups was higher in the combined immunosuppression groups (+ 13.06%) but failed to reach statistical significance (95% CI − 1.71 to + 27.84%, *p* = 0.08; Figs. 2 and 3). Data was incorporated into a random effects model as the test for heterogeneity was significant (*p* < 0.001).

### Statins

Within the predefined period, only one study has evaluated the effect of statin treatment on EMB-proven myocarditis [28]. Wojnicz et al. used 40 mg atorvastatin per day and found a significantly higher LVEF after 6 months in the treatment group (*n* = 34) with a significant increase from 27 ± 7 to 34 ± 8% compared to a non-significant increase in the control group (*n* = 37) of 29 ± 7 to 30 ± 6%. Furthermore, the authors found a significant improvement of NYHA functional class in the treatment group compared to controls [28].

### Immunoglobulins and immunoadsorption

To date, one study has been conducted investigating IVIG infusion alone in myocarditis. Kishimoto et al. treated patients with myocarditis with 1–2 g/kg IVIG over 2 days and found a significant survival benefit after 60 days of follow-up. Interestingly, the IVIG-treated patients did not show an increased LVEF as compared to the conventionally treated control group [30]. Staudt et al. evaluated the effect of immunoadsorption and subsequent IVIG substitution in patients with myocarditis. In this trial, patients were randomized for IA therapy and subsequent IgG substitution at 1-month intervals until month 3. There was a 10 to 11% higher LVEF and a significantly better NYHA functional class in patients treated with immunoadsorption compared to controls. Furthermore, the authors demonstrated that CD3+ T cells decreased significantly in the myocardium in patients treated with immunoadsorption (from 5.7 ± 0.8 to 2.9 ± 0.5 cells/mm^2^, mean ± SEM, *p* < 0.01, compared to baseline and controls) [27].

### Interferon and thymomodulin

Miric et al. separately investigated the effect of interferon-α or thymic hormones in patients with dilated heart muscle disease and myocarditis. They found a significantly greater improvement of the LVEF in both treatment groups of 3 to 4% compared to the control group. Moreover, NYHA functional class improved in 11 of 14 patients in the interferon group and in 8 of 13 patients in the thymomodulin group in contrast to 5 of 13 patients in the control group. Regarding resolution of inflammation, the authors reported that in 5 out of 5 patients that received interferon-α, in 4 out of 5 patients that received thymomodulin, and in 2 out of 5 patients receiving conventional treatment myocarditis, inflammation had resolved in the follow-up biopsy [25].

## Discussion

To date, treatment of myocarditis and DCM largely focuses on supportive care with guideline-directed treatment of heart failure and arrhythmia [3]. Although modulation of the immune response in disease entities caused by maladaptive hyperimmune responses to infectious triggers has been considered potentially beneficial, the limited availability of randomized controlled trials testing this hypothesis reflects the immature state of this body of literature [31]. In the course of this systematic review, we could identify nine trials that assessed the effect of immunomodulatory or immunosuppressive therapies on surrogate endpoints in patients with EMB-proven myocarditis (Fig. 3). We identified prednisone, immunosuppressive combination therapy, interferone/thymomodulin, statins, immunoadsorption, and IVIG as tested treatment approaches. Fours studies provided survival or event-free survival data [23, 24, 26, 30]. Only one study [30] found a potential survival benefit in their study group. Due to the fact that all of these studies investigated different treatment strategies (immunosuppressive combination therapy, IVIG, statins, and prednisone), a meta-analysis was not feasible. To assess a potential pooled effect, we focused on changes in LVEF as a surrogate endpoint. With three studies assessing the effect of combination therapy with immunosuppressants and corticosteroids on LVEF, immunosuppression was identified as the most investigated treatment strategy. Overall, the studies reported conflicting results. Two of three studies found consistently better LVEF and NYHA functional class over time in the treatment group compared to the control group. Both studies revealing beneficial effects used azathioprine as immunosuppressant, whereas the failing study used either azathioprine or cyclosporine [24, 26, 29]. Interestingly, the upregulation of HLA expression in the myocardium was found to be a useful criterion to identify patients that might benefit from the combined therapy [26]. The meta-analysis showed a non-significant effect of combined immunosuppression with a pooled difference of 13.06% for the increase of LVEF over the study period (Fig. 2). Two studies found no benefit from corticosteroid treatment alone in patients with DCM or myocarditis [22, 23]. Common minor side effects such as increased weight were reported in patients treated with corticosteroids [22, 26]. Immunoadsorption and subsequent IVIG substitution were associated with a significantly greater improvement in LVEF compared to the control group in one study [27]. Additionally, this study found a significantly better NYHA functional class and a significantly decreased CD3+ T cell count in the myocardium in the treatment group [27]. In contrast to these findings, it has been observed that IVIG alone did not significantly improve recovery of surrogate endpoints in the treatment group [30]. One study which assessed the effect of interferon or an interferon-inducing agent found a significantly higher LVEF in the treatment groups compared to controls after 6 months [25].

## Conclusions

Overall, the observations of the analyzed studies point towards the benefit of immunomodulation and immunosuppression in myocarditis but the potentially beneficial effects are solely based on changes in surrogate endpoints. The results for combined immunosuppression are conflicting and the meta-analysis could not show a statistically significant positive effect on LVEF. Statins, interferons, and immunoadsorption showed a positive effect on LVEF, but the relatively low evidence of only one study respectively does not permit a general recommendation. When considering the ratio between risk/cost and benefit, one may speculate that the following two therapeutic scenarios may be the most promising in the future: (a) the use of immunoadsorption or combined immunosuppression, consuming large resources, in selected high-risk patients (e.g., by EMB) and (b) the use of statins, associated with only limited costs and few side effects, in a less strictly selected patient population with proven non-ischemic cardiomyopathy and only moderately reduced LVEF. In line with this assumption, the highly lethal etiologies of myocarditis (acute necrotizing eosinophilic and giant cell myocarditis) are more commonly treated with immunosuppression. Although not validated by robust randomized controlled data, this treatment approach has shown promising results in case series and might suggest the translation into traditional lymphocytic myocarditis [16, 32–34].

## Limitations

**Fig. 4:**
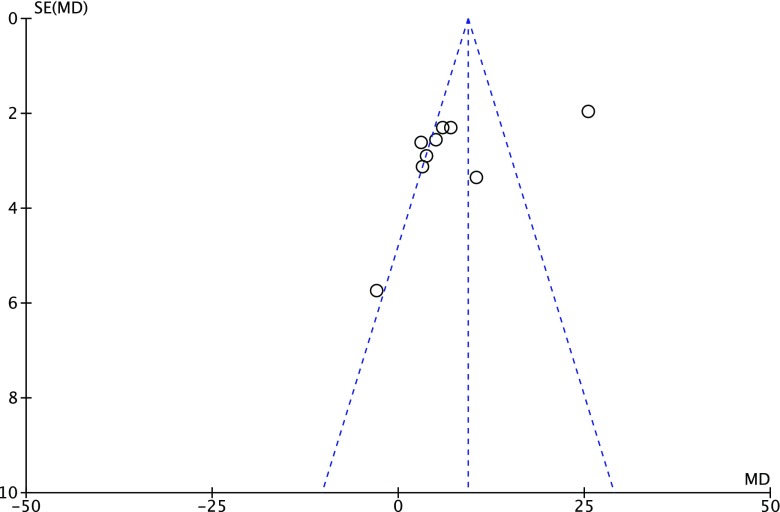
Funnel plot for the assessment of publication bias for studies investigating left ventricular ejection fraction (%)

It must be emphasized that several issues impair the applicability of the results in general population. Firstly and most importantly, the included studies all showed difficulties to obtain well-defined study populations. We observed that in studies that reported the numbers of screened patients, only 10% underwent endomyocardial biopsy (EMB) and only 5% were finally enrolled. These numbers reflect the difficulty to identify patients with proven myocarditis. Especially in immunosuppressive therapy regimens a proper patient selection and valid EMB results are needed as it has been shown that particularly patients with evidence of inflammatory activation in the myocardium benefit from those treatments [26]. Another obstacle restraining from routine use of immunosuppressive therapy is the challenge to differentiate between virus-positive and virus-negative myocarditis. The clinical practice of testing of viral antibody titers owes only a low specificity, as the cardiotropic viruses including coxsackievirus and echovirus certainly dominate, but a myriad of viruses can cause myocarditis. To rely on this approach might result in a non-negligible proportion of false-virus-negative cases. The use of endomyocardial biopsy for the diagnosis of viral genomes in the myocardium might be beneficial to distinguish between virus-positive and virus-negative myocarditis, but its sensitivity for this purpose has not been reported and remains a matter of speculation [35]. Subsuming both clinical routine and the majority of the trials [23–25], [22, 26, 28] struggle to safely exclude viral infections. All results obtained from the performed meta-analysis must be interpreted with caution due to the significant clinical heterogeneity between the included studies. In myocarditis, the evaluation of changes between the treatment group and a control group is of particular importance in this setting, as spontaneous improvement in the majority of patients led to an improvement in many cohorts regardless of the therapeutic agent. Randomization avoided confounding and selection bias during enrolment in treatment groups. An insufficient description of the generation of the allocation sequence and/or concealment of the allocation impaired the quality of all studies. The lack of double blinding was a major limitation in most studies, particularly when assessing endpoints with considerable inter-observer variability such as LVEF measured by echocardiography or when using the subjective reporting of functional status by patient. Selection bias may have occurred as the majority of studies did not report loss to follow-up and/or did not use an intention-to-treat analysis. Furthermore, the inclusion of only biopsy-proven myocarditis per se may lead to a selection bias as it favors the inclusion of more severe cases of myocarditis and is poorly sensitive to detect those patients with transient or focal myocardial inflammation. However, there are no prospective randomized trials that used clinical/radiologic criteria instead of biopsy criteria. No evidence was found for publication bias when searching for unpublished studies or by the use of a funnel plot (Fig. 4).

## Implication for research

Future research should focus on translation of the effects of statins and immunoadsorption on surrogate endpoints to clinical outcome. Therefore, large multi-center trials are required to ensure a sufficient number of endpoints in each treatment group. Furthermore, future studies should try to confirm promising results from single studies suggesting a beneficial effect of thalidomide and interferon. Finally, it may be worth focusing on a specific treatment approach in patients with EMB-proven myocarditis. Regarding methodology, future RCTs should give better descriptions of allocation of treatment, concealment of allocation, and the study flow and should furthermore use double blinding with placebo treatment in the control group. Additionally, as it is not a clinical practice to carry out EMB in all myocarditis patients, RCT’s should also enroll patients with clinically suspected or better imaging-confirmed myocarditis.
